# Studies on the Bioavailability of Deoxynivalenol (DON) and DON Sulfonate (DONS) 1, 2, and 3 in Pigs Fed with Sodium Sulfite-Treated DON-Contaminated Maize

**DOI:** 10.3390/toxins7114622

**Published:** 2015-11-05

**Authors:** Marleen Paulick, Janine Winkler, Susanne Kersten, Dian Schatzmayr, Heidi Elisabeth Schwartz-Zimmermann, Sven Dänicke

**Affiliations:** 1 Institute of Animal Nutrition, Friedrich-Loeffler-Institute (FLI), Federal Research Institute for Animal Health, Bundesallee 50, 38116 Braunschweig, Germany; E-Mails: marleen.paulick@fli.bund.de (M.P.); janine.winkler@fli.bund.de (J.W.); susanne.kersten@fli.bund.de (S.K.); 2 Biomin Holding GmbH, Biomin Research Center, Technopark 1, 3430 Tulln, Austria; E-Mail: dian.schatzmayr@biomin.net; 3 Christian Doppler Laboratory for Mycotoxin Metabolism and Center for Analytical Chemistry, Department for Agrobiotechnology (IFA-Tulln), University of Natural Resources and Life Sciences, Vienna, Konrad Lorenz Str. 20, 3430 Tulln, Austria; E-Mail: heidi.schwartz@biomin.net

**Keywords:** decontamination, sodium sulfite, deoxynivalenol, deoxynivalenol sulfonates, toxicokinetics, bioavailability, pigs

## Abstract

Deoxynivalenol (DON) exposure of pigs might cause serious problems when critical dietary toxin concentrations are exceeded. As DON contamination of agricultural crops cannot be completely prevented, detoxification measures are needed. Wet preservation with sodium sulfite resulted in a significant DON reduction of naturally-contaminated maize in previous experiments. The preserved material had a characteristic DON sulfonates (DONS) pattern. DONS is known to be less toxic than DON but its stability was shown to depend on pH, which gives rise to the question if a back-conversion to DON occurs *in vivo*. Therefore, the toxicokinetics and bioavailability of DON and DONS were studied in pigs. After the administration of a single oral or intravenous bolus of DON or DONS, serial blood samples were collected and subsequently analyzed. DONS was not detectable after oral administration of DONS mixtures. The results showed further that the bioavailability of DONS as DON in pigs fed maize preserved wet with sodium sulfite was significantly decreased compared to untreated control maize (DON), indicating that DONS obviously did not convert back to DON to a large extent *in vivo*. Moreover, the fact that DONS was not detectable in systemic blood requires further investigations regarding their ingestive and/or metabolic fate.

## 1. Introduction

The occurrence of the mycotoxin deoxynivalenol (DON) in cereals owing to mold infestation by *Fusarium* ssp. cannot be completely avoided in the temperate climate zone. The use of contaminated feed is regulated by the European Commission [1] in order to protect farm animals from health-compromising mycotoxin effects. Pigs are among the most sensitive species. Effects of DON intoxication are: reduced feed intake up to feed refusal, salivation, sickness, and also vomiting. Due to these adverse effects, performance in exposed animals decreases [2] when the guidance value of 0.9 mg DON/kg feed for pigs is exceeded. A possibility to use contaminated cereals without adverse effects on health and performance is decontamination [3]. Previous studies demonstrated that sulfur-containing compounds can be applied to decontaminate DON in feed [4,5,6,7]. A preservation experiment with DON-contaminated maize treated with sodium sulfite (Na_2_SO_3_) demonstrated a significant DON reduction by 100% due to the addition of 10 g Na_2_SO_3_ per kg of maize [8]. New reaction products are formed as a result of the reaction between DON and the sulfur reagents as described by Schwartz *et al.* [9]. These metabolites, the so-called DON sulfonates (DONS) 1, 2, and 3, are characterized, besides other structural modifications, by the loss of the double bond (C9=C10) and the addition of a sulfonate group at C10. DONS 1, characterized by the absence of the epoxide group, is the most stable form over a pH range of 2–10. DONS 2, characterized by a hemiketal, is stable at pH 2–7 for 24 h. At pH values of 8–10, back-formation to DON can be observed. The DONS 3 compound exists as a mixture of two compounds at a roughly equal ratio. Both a hemiketal- and keto-form occur in equilibrium and DONS 3 is predominantly formed under acidic conditions. Furthermore, DONS 3 is the least stable form and converts to DON, DONS 1, and 2 at neutral and alkaline pH, as well as at longer storage time. The concentration-time profiles of DONS 1, 2, and 3 during a 79-day preservation period in the presence of Na_2_SO_3_ under wet conditions were described recently for DON-contaminated maize meal (MM) and unground maize kernels (MK) [8]. In this experiment, the pH of the treated material was acidic and averaged 4.7 due to the addition of propionic acid aimed at avoiding microbial spoilage. In this case, next to the rapid DON reduction, a concomitant pronounced formation of DONS 3 took place. However, in the course of the preservation time, DONS 3 decreased continuously. In contrast, DONS 1 increased to a small extent and DONS 2 was enhanced substantially.

Based on these pronounced time- and pH-dependent alterations in the pattern of DONS, including the re-formation of DON, the question arises whether the pH-fluctuations within the digestive tract and the physiological blood pH-value of 7.4 contribute to further changes in these profiles. An overwhelming re-formation of DON from DONS would question the wet preservation of DON-contaminated feed with Na_2_SO_3_ as a suitable decontamination measure in general.

In order to answer these questions, the plasma kinetics of DON and DONS were examined with administration of a single intravenous (IV) or oral (po) bolus from either pure standards (DON_iv_, DONS_iv_, DONS_po_), from naturally-contaminated maize, either not treated (NDON), dry supplemented with Na_2_SO_3_ (SDON), or wet preserved with Na_2_SO_3_ for 37 (MM_37_, MK_37_) and 79 (MM_79_, MK_79_) days.

## 2. Results

### 2.1. Clinical Symptoms

Clinical symptoms occurring after intravenous application of 50 µg DON/kg BW (DON_iv_) were retching and vomiting between three and nine times within a few minutes (6–16 min). Twenty-five minutes after the DON bolus, no more emesis appeared. In contrast, neither pigs dosed intravenously with DONS_iv_ nor exposed orally to any of the tested variants showed any clinical signs.

### 2.2. Intravenous Application of DON (DON_iv_)

**Figure 1:**
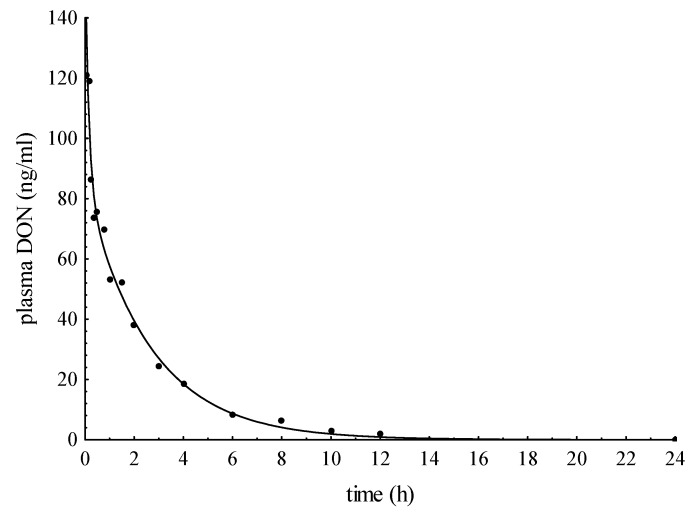
Plasma concentration-time curve after intravenous application of 50 µg DON/kg BW to pig IV5.

The plasma concentration data from five intravenously dosed pigs were fitted to the bi-exponential regression (Equation (2)) corresponding to a two-compartment model. In Figure 1, an exemplary fitted curve is shown together with the individually-analyzed plasma DON concentrations indicating the typical course after intravenous application. In Table 1 the estimated values, as well as derived toxicokinetic parameters were summarized. The mean half-life (t_1/2α_) for distribution amounted to 0.09 ± 0.05 h and demonstrated the immediate availability of DON in the systemic cycle. The slower elimination half-life (t_1/2β_) was, on average, 1.98 ± 0.09 h. The mean apparent volume of distribution (V_d(area)_) was 0.61 ± 0.09 L/kg. The plasma clearance averaged 3.61 ± 0.38 mL/kg min.

**Table 1:** Toxicokinetic parameters of pigs after intravenous injection of DON_iv_.

Animal	IV 1	IV 2	IV 3	IV 4	IV 5	Mean	±S.D.
**BW (kg)**	50.9	52.1	49.0	44.4	45.7	48.4	2.7
**DON (µg/kg BW)**	50.0	50.0	50.0	50.0	50.0		
**A (ng/mL)**	136.4	624.9	121.9	56.2	74.5	202.8	194.6
**α (1/h)**	7.35	18.74	13.71	3.88	6.29	9.99	4.97
**B (ng/mL)**	96.5	83.6	73.5	64.5	84.1	80.5	9.9
**β (1/h)**	0.36	0.33	0.35	0.34	0.38	0.35	0.02
**t_1/2α_ (h)**	0.09	0.04	0.05	0.18	0.11	0.09	0.05
**t_1/2β_ (h)**	1.91	2.12	1.98	2.05	1.83	1.98	0.09
**V_d(area)_ (L/kg)**	0.52	0.67	0.68	0.72	0.53	0.61	0.09
**Cl (mL/kg min)**	3.15	3.66	3.93	4.04	3.33	3.61	0.38
**AUC (ng h/mL)**	264.2	227.8	212.0	206.3	250.0	232.0	20.2
**RSD (ng/mL)**	5.70	7.44	4.27	7.09	6.05	6.11	1.02
***R*^2^**	0.96	0.87	0.97	0.97	0.98	0.95	0.04

BW = body weight, *A* + *B* = initial concentration at *t* = 0, α and β = rate constants of distribution or elimination phase, *t* = time (h), t_1/2α_ and t_1/2β_ (h) = half-life of distribution or elimination, V_d(area)_ (L/kg) = apparent volume of distribution, Cl (mL/kg min) = plasma clearance, AUC (ng·h/mL) = area under the curve, RSD (ng/mL) = residual standard deviation, *R*^2^ = coefficient of determination, S.D. = standard deviation

### 2.3. Intravenous Administration of DONS (DONS_iv_)

**Figure 2:**
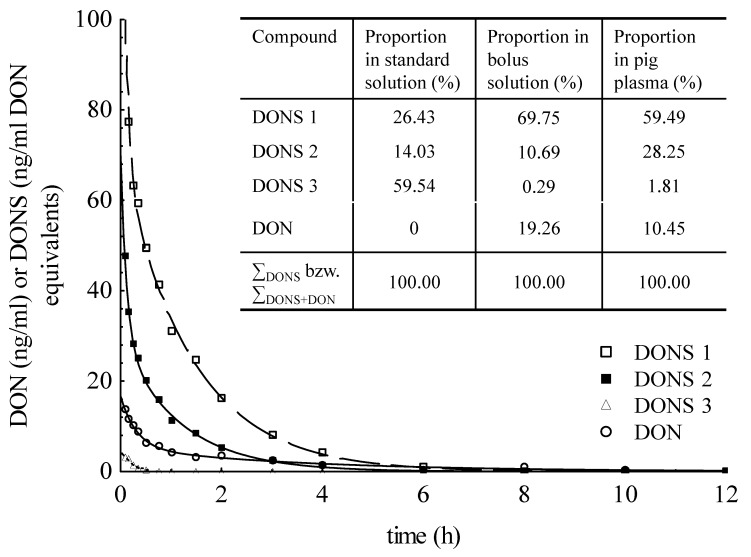
Plasma kinetic of pigs (means) after intravenous application of 50 µg DONS mixture per kg BW as well as DONS ratio in injected bolus (inserted table).

The mean plasma concentrations of each DON sulfonate are shown in Figure 2. Additionally, the proportions of DONS 1, 2 and 3 in standard as well as bolus solutions were compared in the little inserted table (Figure 2). Only DONS 1 and 2, as well as DON were detectable in bolus solution despite the marked presence of DONS 3 in the standard solution. The comparison of pH values in standard and bolus solution revealed 1.56 and 2.66. Derived from the plasma concentrations after injection, it could be observed that DONS 1 and 2 were stable in plasma with peak concentrations (mean of *A* + *B*) of 171.4 ng/mL and 79.9 ng/mL. In contrast, DONS 3 was only detected to a very small extent (LOD < *x* < LOQ) and the peak concentration achieved 2.39 ng/mL DON equivalents (mean). In the following, concentration data of DONS in ng/mL were already converted in ng/mL DON equivalents because of the higher molecular mass of DONS compared to DON. Twenty minutes after bolus, no more DONS 3 could be determined whereas the plasma clearance of DONS 1 and 2 amounted to 5.0 ± 0.4 mL/kg min and 9.9 ± 1.1 mL/kg min (Table 2). The area under the curve (AUC) was 168.2 ± 13.9 ng·h/mL and 85.8 ± 10.9 ng·h/mL for DONS 1 and 2, respectively.

The immediate distribution in plasma after IV injection (t_1/2_α) was characterized by half-lives of 0.1 ± 0.05 h and 0.1 ± 0.1 h for DONS 1 and 2, respectively. The elimination half-life was around 1.0 h for both substances. Regarding the percentage composition of standard and bolus solution, the differences in DONS pattern as well as the occurrence of DON suggested that the compounds were not stable during the preparation process. In addition to DONS, DON was also detected with a mean maximum concentration (*A* + *B*) of 17.33 ng/mL (Table 3). The apparent volume of distribution was on average 4.91 ± 0.77 L/kg and the plasma clearance amounted to 21.46 ± 4.13 mL/kg·min. The mean distribution half-life (t_1/2α_) was on average 0.23 ± 0.10 h and the slower elimination half-life (t_1/2β_) was 2.74 ± 0.71 h.

### 2.4. Untreated DON-Containing Control, either Untreated (NDON) or Dry Treated (SDON) with Sodium Sulfite

**Figure 3:**
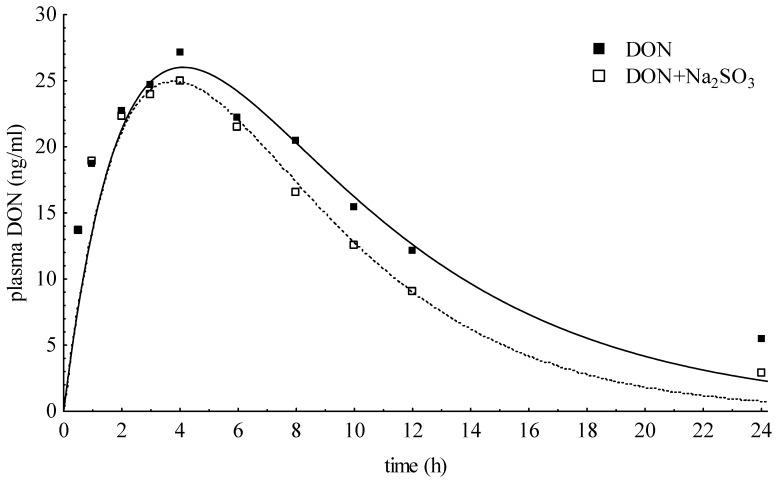
Comparison of plasma concentration-time curves after oral administration of DON containing feed with (SDON) or without (NDON) supplementation of sodium sulfite.

Six pigs fed with an oral single dose of DON contaminated feed (6 mg DON/kg feed) served as the negative DON control group (NDON). A further six pigs were fed with the negative control diet but additionally supplemented with (dry) sodium sulfite immediately before feed consumption (SDON). No signs of toxic effects were observed in either group. In Figure 3, DON plasma concentrations of NDON and SDON (means) were compared. The area under the curve (AUC) of SDON was about 13.9% lower than NDON. The mean half-lives for distribution (t_1/2α_) for NDON and SDON amounted 2.50 ± 3.27 h and 1.02 ± 0.72 h followed by slower elimination half-lives of 6.05 ± 1.92 h and 5.63 ± 0.94 h, respectively (Table 4). The apparent volume of distribution ranged between 1.13 and 2.66 L/kg for both. The clearance of plasma covered a range from 6.35 to 17.36 mL/kg·min for NDON and 12.62 to 17.53 mL/kg·min for SDON and, therewith, the clearance was nearly two to five times greater than in the DON_iv_ group.

**Table 2:** Toxicokinetic parameters for DONS 1 and 2 after intravenous injection of DONS_iv_.

Animal	IV6		IV7		IV8		IV9		IV10		IV11		Mean		±S.D.	
Compound	DONS 1	DONS 2	DONS 1	DONS 2	DONS 1	DONS 2	DONS 1	DONS 2	DONS 1	DONS 2	DONS 1	DONS 2	DONS 1	DONS 2	DONS 1	DONS 2
**BW (kg)**	47.6		41.6		42.2		40.9		42.2		42.9		42.9		2.2	
**DONS (µg/kg BW)**	50.0		50.0		50.0		50.0		50.0		50.0		50.0		0.0	
**A (ng/mL)**	86.78	43.30	60.09	34.43	257.42	126.49	79.19	42.60	80.20	46.01	61.27	23.41	104.2	52.7	69.2	33.8
**α (1/h)**	11.35	9.99	4.94	4.03	20.41	17.39	4.55	4.41	10.96	12.01	13.56	4.93	11.0	8.8	5.4	4.9
**B (ng/mL)**	74.30	29.84	61.46	19.94	75.58	34.75	67.17	25.99	69.69	32.87	55.10	20.05	67.2	27.2	7.1	5.8
**β (1/h)**	0.80	0.93	0.59	0.59	0.72	0.90	0.70	0.76	0.71	0.90	0.72	0.70	0.7	0.8	0.1	0.1
**t_1/2α_ (h)**	0.06	0.07	0.14	0.17	0.03	0.04	0.15	0.16	0.06	0.06	0.05	0.14	0.1	0.1	0.05	0.1
**t_1/2β_ (h)**	0.87	0.74	1.18	1.17	0.96	0.77	0.99	0.91	0.98	0.77	0.96	0.99	1.0	0.9	0.1	0.2
**V_d(area)_ (L/kg)**	0.35	0.67	0.51	1.08	0.37	0.53	0.44	0.69	0.42	0.68	0.48	0.96	0.4	0.8	0.1	0.2
**Cl (mL/kg·min)**	4.64	10.41	4.97	10.67	4.44	7.88	5.10	8.81	4.97	10.22	5.82	11.18	5.0	9.9	0.4	1.1
**AUC (ng·h/mL)**	179.7	80.1	167.6	78.1	187.8	105.8	163.4	94.6	167.8	81.5	143.2	74.6	168.2	85.8	13.9	10.9
**RSD (ng/mL)**	1.37	0.39	5.00	1.70	1.98	0.68	5.98	2.71	0.89	0.50	0.82	0.42	2.7	1.1	2.0	0.9
***R*^2^**	1.00	1.00	0.99	0.99	1.00	1.00	0.99	0.99	1.00	1.00	1.00	1.00	1.0	1.0	0.0	0.0

BW = body weight, *A* + *B* = initial concentration at *t* = 0, α and β = rate constants of distribution or elimination phase, *t* = time (h), t_1/2α_ and t_1/2β_ (h) = half-life of distribution or elimination, V_d(area)_ (L/kg) = apparent volume of distribution, Cl (mL/kg min) = plasma clearance, AUC (ng·h/mL) = area under the curve, RSD (ng/mL) = residual standard deviation, *R*^2^ = coefficient of determination; S.D. = standard deviation.

**Table 3:** Toxicokinetic parameters of DON in plasma of pigs after IV application of DONSiv.

Animal	IV6	IV7	IV8	IV9	IV10	IV11	Mean	±S.D.
**BW (kg)**	47.6	41.6	42.2	40.9	42.2	42.9	42.9	2.2
**DON (µg/kg BW)**	0.0	0.0	0.0	0.0	0.0	0.0	0.0	0.0
**DONS (µg/kg BW)**	50.0	50.0	50.0	50.0	50.0	50.0	50.0	0.0
**A (ng/mL)**	12.44	8.40	17.13	12.74	12.50	10.51	12.29	2.65
**α (1/h)**	5.02	3.23	4.83	1.72	3.99	2.25	3.51	1.23
**B (ng/mL)**	7.13	5.49	5.08	2.91	6.48	3.17	5.04	1.56
**β (1/h)**	0.32	0.22	0.34	0.17	0.29	0.27	0.27	0.06
**t_1/2α_ (h)**	0.14	0.22	0.14	0.40	0.17	0.31	0.23	0.10
**t_1/2β_ (h)**	2.16	3.14	2.02	4.11	2.43	2.57	2.74	0.71
**V_d(area)_ (L/kg)**	5.40	5.64	3.45	5.39	4.37	5.23	4.91	0.77
**Cl (mL/kg·min)**	28.81	20.76	19.77	15.12	20.79	23.54	21.46	4.13
**AUC (ng·h/mL)**	28.92	40.14	42.16	55.11	40.09	35.40	40.31	7.92
**F (%)**	12.5	17.3	18.2	23.8	17.3	15.3	17.37	3.41
**RSD (ng/mL)**	0.9	0.4	0.8	1.6	1.0	1.1	1.0	0.36
***R*^2^**	0.98	0.99	0.99	0.96	0.98	0.97	0.98	0.01

BW = body weight, *A* + *B* = initial concentration at *t* = 0, α and β = rate constants of distribution or elimination phase, *t* = time (h), t_1/2α_ and t_1/2β_ (h) = half-life of distribution or elimination, V_d(area)_ (L/kg) = apparent volume of distribution, Cl (mL/kg min) = plasma clearance, AUC (ng·h/mL) = area under the curve, F (%) = release rate of DON from DONS, RSD (ng/mL) = residual standard deviation, *R*^2^ = coefficient of determination; S.D. = standard deviation.

**Table 4:** Toxicokinetic parameters of oral administration of the negative DON control (NDON) as well as the supplemented DON control (with dry Na_2_SO_3_; SDON) diet.

**NDON**								
**Animal**	O-1	O-2	O-3	O-4	O-5	O-6	Mean	±S.D.
**BW (kg)**	31.0	43.6	39.2	43.4	38.0	53.0	41.4	6.7
**DON (µg/kg BW)**	97	69	77	69	79	57	75	12
***k_a_* (1/h)**	0.65	1.13	0.62	0.48	0.07	0.72	0.61	0.31
***k_el_* (1/h)**	0.13	0.13	0.10	0.16	0.07	0.16	0.12	0.03
**C_0_ (ng/mL)**	49.72	33.11	47.78	62.20	64.64	31.95	48.23	12.65
**C_max_ (ng/mL)**	33.54	25.01	33.79	35.74	23.78	20.69	28.76	5.78
**t_max_ (h)**	3.12	2.17	3.56	3.42	14.13	2.69	4.85	4.18
**t_1/2α_ (h)**	1.06	0.61	1.13	1.45	9.80	0.97	2.50	3.27
**t_1/2β_ (h)**	5.50	5.35	7.12	4.28	9.80	4.29	6.05	1.92
**V_d(area)_ (L/kg)**	1.95	2.08	1.61	1.15	1.50	1.79	1.68	0.31
**Cl (mL/kg·min)**	14.76	16.14	9.39	11.21	6.35	17.36	12.53	3.89
**AUC (ng·h/mL)**	355.77	244.09	439.42	363.06	468.99	186.91	343.04	99.90
**F (%)**	79.2	76.4	123.7	113.2	128.0	71.2	98.6	23.6
**RSD (ng/mL)**	2.61	2.25	3.32	3.52	3.70	1.52	2.82	0.77
***R*^2^**	0.98	0.97	0.97	0.97	0.93	0.98	0.97	0.02
**SDON**								
**Animal**	O-7	O-8	O-9	O-10	O-11	O-12	Mean	±S.D.
**BW (kg)**	35.8	47.4	37.8	40.8	34.0	46.9	40.5	5.2
**DON (µg/kg BW)**	57	63	79	74	88	64	71	11
***k_a_* (1/h)**	0.93	1.72	0.79	0.62	0.27	1.60	0.99	0.52
***k_el_* (1/h)**	0.12	0.12	0.12	0.11	0.19	0.11	0.13	0.03
**C_0_ (ng/mL)**	28.49	36.23	37.01	27.43	78.97	29.10	39.54	18.03
**C_max_ (ng/mL)**	21.15	29.70	26.49	18.91	34.21	23.86	25.72	5.15
**t_max_ (h)**	2.56	1.67	2.84	3.38	4.31	1.79	2.76	0.91
**t_1/2α_ (h)**	0.75	0.40	0.88	1.11	2.53	0.43	1.02	0.72
**t_1/2β_ (h)**	5.95	5.83	5.88	6.30	3.57	6.25	5.63	0.94
**V_d(area)_ (L/kg)**	2.00	1.77	2.16	2.66	1.13	2.17	1.98	0.47
**Cl (mL/kg·min)**	13.98	12.62	15.27	17.53	14.99	14.44	14.80	1.49
**AUC (ng·h/mL)**	232.91	303.89	303.89	259.48	447.72	224.61	295.42	74.78
**F (%)**	88.6	103.5	82.5	76.0	109.3	75.7	89.3	13.0
**RSD (ng/mL)**	0.95	2.63	2.58	1.62	2.87	1.48	2.02	0.71
***R*^2^**	0.99	0.98	0.97	0.98	0.98	0.99	0.98	0.01

BW = body weight, *c_t_* = initial concentration at time *t*, *k_a_* and *k_el_* = constants of absorption or elimination, t_1/2α_ and t_1/2β_ (h) = half-life of distribution or elimination, V_d(area)_ (L/kg) = apparent volume of distribution, Cl (mL/kg min) = plasma clearance, AUC (ng h/mL) = area under the curve, F (%) = bioavailability of DON = (AUC_po_/AUC_iv_) × 100, RSD (ng/mL) = residual standard deviation, *R*^2^ = coefficient of determination, S.D. = standard deviation.

### 2.5. Oral Administration of DONS Mix to Basal Diet (DONS_po_)

**Figure 4:**
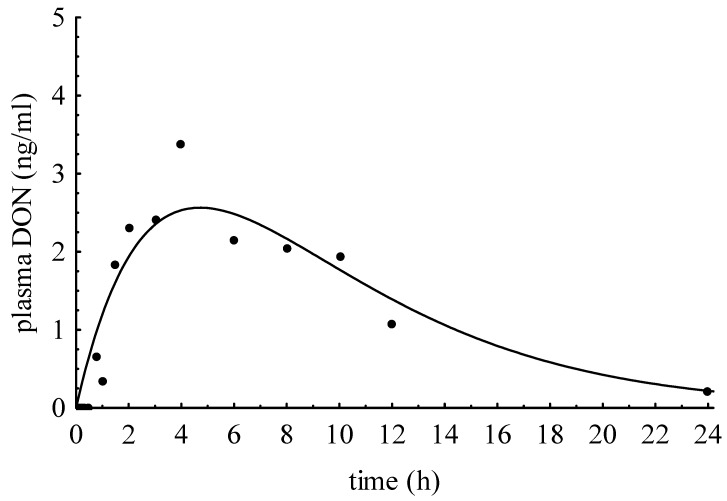
DON plasma concentrations after oral administration of 100 µg DONS per kg BW.

The addition of 100 µg DONS mix/kg BW to uncontaminated maize diet should provide further information on the kinetics and metabolism of DON sulfonates after oral exposure. As a result, no DONS compound could be detected in plasma samples but DON appeared in a typical time-dependent manner observable after oral DON administration (Figure 4). The peak concentration (c_max_) of 2.56 ng/mL was achieved after 4.71 h (t_max_). The area under the curve (AUC) was 4.30 ng/h mL (mean) and the plasma clearance of DON amounted to 17.41 mL/kg min.

### 2.6. Wet Preserved, Sodium Sulfite-Treated Variants (MK_37_, MM_37_, MK_79_, MM_79_)

**Figure 5:**
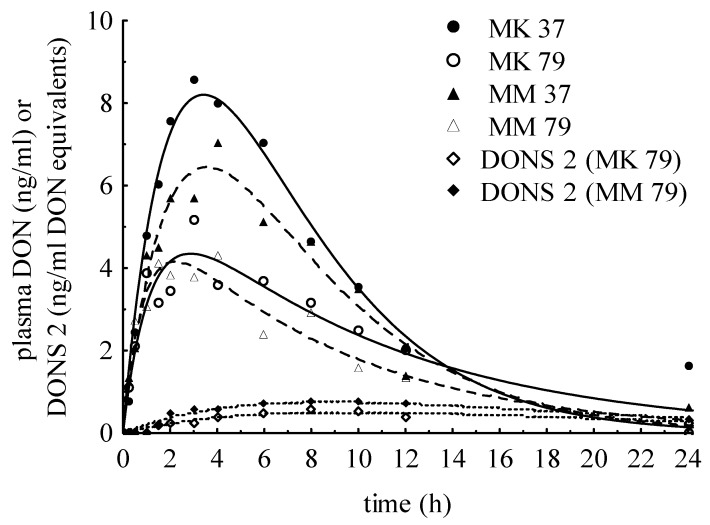
Plasma concentration-time curves after feeding with MK_37_/MM_37_ and MK_79_/MM_79_ (means) illustrated the DON kinetic as well as DONS 2 also detected in minor extent after MK_79_/MM_79_.

Although only DONS was detected in sodium sulfite wet-treated MM and MK, DON was detected in plasma samples but at much lower levels compared to NDON group. The DONS exposure ranged between 67.78 to 95.59 µg/kg BW, but no DONS 1, was detected in any plasma samples and tiny amounts of DONS 2 could be determined in plasma samples of variants MK_79_ and MM_79_ (LOD < *x* < LOQ). Maximum concentrations were only 1.10 and 0.82 ng/mL DON equivalents. The mean peak concentration of DON amounted to 7.24 ng/mL fed with MK_37_ or MM_37_ (Table 5) as well as 4.33 ng/mL for MK_79_ or MM_79_ (Table 6). In Figure 5, plasma concentration-time curves of pigs fed different preserved variants were compared. Additionally, DONS 2 concentrations after oral administration of MK_79_ and MM_79_ are shown. The area under the curve (AUC, means) for MK_37_/MM_37_ and MK_79_/MM_79_ were reduced by 89.2% and 94.5% in comparison to the negative DON control group. The plasma clearance of all tested pigs was 6.11 ± 2.06 mL/kg min and was only a half of the DON oral group.

**Table 5:** Kinetic parameters of pigs after oral administration of wet preserved diets (MK_37_ and MM_37_). It is important to note that the parameters *k_a_* to *R*^2^ refer to the course of DON concentration in plasma, not to DONS.

Kinetic parameters	MK_37_						MM_37_					
**Animal**	O-13	O-14	O-15	O-16	Mean	±S.D.	O-17	O-18	O-19	O-20	Mean	±S.D.
**BW (kg)**	37.3	36.0	37.3	38.0	37.2	0.7	46.0	47.2	44.1	38.1	43.9	3.5
**DON (µg/kg BW)**	0	0	0	0	0	0	0	0	0	0	0	0
**DONS (µg/kg BW)**	87.6	91	88	86	88	2	79	77	83	96	84	7
***k_a_* (1/h)**	0.50	0.33	0.35	0.31	0.37	0.07	0.44	0.25	0.54	0.74	0.49	0.18
***k_el_* (1/h)**	0.20	0.23	0.21	0.31	0.24	0.04	0.20	0.25	0.15	0.10	0.18	0.06
**C_0_ (ng/mL)**	15.84	21.36	17.32	18.18	18.18	2.02	15.15	16.97	8.77	7.99	12.22	3.90
**C_max_ (ng/mL)**	8.65	9.31	8.00	6.69	8.16	0.97	7.86	6.24	5.34	5.85	6.32	0.94
**t_max_ (h)**	3.10	3.57	3.62	3.22	3.38	0.22	3.27	3.94	3.26	3.13	3.40	0.32
**t_1/2α_ (h)**	1.40	2.07	1.98	2.23	1.92	0.31	1.57	2.73	1.28	0.94	1.63	0.67
**t_1/2β_ (h)**	3.55	2.98	3.25	2.23	3.00	0.49	3.46	2.73	4.55	6.96	4.43	1.60
**V_d(area)_ (L/kg)**	1.01	0.39	0.49	0.35	0.56	0.26	0.58	0.40	0.62	0.55	0.54	0.08
**Cl (mL/kg·min)**	11.82	5.39	6.31	6.56	7.52	2.52	6.93	6.06	5.70	3.30	5.50	1.35
**AUC (ng·h/mL)**	32.49	23.66	40.24	57.34	38.43	12.39	23.40	28.46	57.58	34.46	35.98	13.07
**F (%)**	8.0	5.6	9.9	14.4	9.5	3.2	6.4	8.0	15.0	7.8	9.3	3.4
**RSD (ng/mL)**	0.92	0.92	1.60	1.07	1.13	0.28	1.32	1.20	1.16	1.17	1.21	0.06
***R*^2^**	0.97	0.93	0.90	0.95	0.94	0.03	0.93	0.91	0.90	0.89	0.91	0.01

BW = body weight, *c_t_* = initial concentration at time *t*, *k_a_* and *k_el_* = constants of absorption or elimination, t_1/2α_ and t_1/2β_ (h) = half-life of distribution or elimination, V_d(area)_ (L/kg) = apparent volume of distribution, Cl (mL/kg min) = plasma clearance, AUC (ng·h/mL) = area under the curve, F (%) = release rate of DON from DONS = (DON − AUC_po_/DON − AUC_DONSiv_) × 100, RSD (ng/mL) = residual standard deviation, *R*^2^ = coefficient of determination, S.D. = standard deviation.

**Table 6:** Kinetic parameters of pigs after oral administration of wet preserved diets (MK_79_ and MM_79_). It is important to note that the parameters *k_a_* to *R*^2^ refer to the course of DON concentration in plasma, not to DONS.

Kinetic parameters	MK_79_							MM_79_						
**Animal**	O-21	O-22	O-23	O-24	O-25	Mean	±S.D.	O-26	O-27	O-28	O-29	O-30	Mean	±S.D.
**BW (kg)**	48.1	49.3	46.2	40.2	41.5	45.1	3.6	40.2	42.2	49.5	50.7	50.9	46.7	4.6
**DON (µg/kg BW)**	0	0	0	0	0	0	0	0	0	0	0	0	0	0
**DONS (µg/kg BW)**	68	68	72	82	81	74	6	90	86	73	71	71	78	8
***k_a_* (1/h)**	0.41	0.41	1.27	0.80	1.41	0.86	0.42	1.16	3.41	0.71	0.97	0.90	1.43	1.00
***k_el_* (1/h)**	0.20	0.11	0.08	0.07	0.14	0.12	0.05	0.17	0.10	0.11	0.18	0.07	0.13	0.04
**C_0_ (ng/mL)**	11.68	8.18	4.13	4.48	5.85	6.86	2.80	6.13	4.53	5.81	7.46	4.05	5.60	1.21
**C_max_ (ng/mL)**	5.92	4.99	3.42	3.51	4.51	4.47	0.94	4.42	4.09	4.13	5.09	3.24	4.19	0.60
**t_max_ (h)**	3.44	4.32	2.31	3.28	1.80	3.03	0.89	1.95	1.08	3.10	2.14	3.03	2.26	0.75
**t_1/2α_ (h)**	1.69	1.69	0.55	0.87	0.49	1.06	0.53	0.60	0.20	0.97	0.72	0.77	0.65	0.26
**t_1/2β_ (h)**	3.51	6.08	8.48	9.28	4.79	6.43	2.18	4.14	7.25	6.31	3.89	9.48	6.73	2.00
**V_d(area)_ (L/kg)**	0.51	0.62	0.84	0.70	0.79	0.69	0.12	0.76	1.28	0.70	0.86	1.06	0.93	0.21
**Cl (mL/kg·min)**	6.04	4.26	4.13	3.16	6.89	4.90	1.36	7.60	7.32	4.60	9.23	4.64	6.68	1.80
**AUC (ng·h/mL)**	19.83	8.16	16.82	6.63	23.93	15.07	6.68	9.22	46.85	13.09	31.48	11.08	22.34	14.62
**F (%)**	6.2	2.6	5.0	1.8	6.4	4.4	1.9	2.2	11.8	3.9	9.5	3.4	6.2	3.8
**RSD (ng/mL)**	0.91	1.54	0.22	0.92	1.03	0.92	0.42	1.25	1.26	1.18	1.13	0.51	1.07	0.28
***R*^2^**	0.94	0.81	0.99	0.86	0.90	0.90	0.06	0.87	0.80	0.84	0.90	0.94	0.87	0.05

BW = body weight, *c_t_* = initial concentration at time *t*, *k_a_* and *k_el_* = constants of absorption or elimination, t_1/2α_ and t_1/2β_ (h) = half-life of distribution or elimination, V_d(area)_ (L/kg) = apparent volume of distribution, Cl (mL/kg·min) = plasma clearance, AUC (ng·h/mL) = area under the curve, F (%) = release rate of DON from DONS = (DON – AUC_po_/DON − AUC_DONSiv_) × 100, RSD (ng/mL) = residual standard deviation, *R*^2^ = coefficient of determination, S.D. = standard deviation.

### 2.7. Bioavailability and Further Toxicokinetic Parameters

After IV injection of DON, a 100% systemic bioavailability is assumed. In comparison, the DON availability after oral administration of NDON was 98.6% ± 23.6%. The supplementation of dry sodium sulfite (SDON) reduced the availability to 89.26% ± 12.98%. Due to the wet preservation treatment of contaminated maize with sodium sulfite, the bioavailability F (%) could be decreased significantly (*p* > 0.001; Table 7). Differences between preserved variants used were marginal and ranged between 5.27% to 9.37% for MK_37_/MM_37_ and MK_79_/MM_79_, respectively. The reduction of the bioavailability compared to oral administration of DON (NDON) amounted to approximately 90%. After IV administration of DONS, DON occurred in plasma samples which might be the result of the changes in proportions from standard to bolus solution. The determination of DON after preparing the bolus solution substantiated this assumption. Hence, the detection of DON after oral exposure of DONS_po_ added to the diet was not surprising. In these cases, bioavailability is defined as the release of DON from DONS. This release rate of DON from DONS was about 17.9% ± 4.5%.

Further statistical tests demonstrated that no significant differences existed between NDON and SDON, as well as among preserved variants themselves (Table 7). However, as expected, NDON and SDON differentiated significantly (*p* < 0.05) from preserved variants in parameters c_max_, V_*d(area)*_, Cl and AUC. Other kinetic parameters t_max_, t_1/2__α_ and t_1/2__β_ showed no significance whereby the half-life of elimination t_1/2β_ of MK_37_ and MM_37_ tended to be shorter than MK_79_ and MM_79_.

**Table 7:** Analysis of variance of selected toxicokinetic parameters after oral administration of different variants.

variants	*n*	C_max_ (ng/mL)	t_max_ (h)	t_1/2α_ (h)	t_1/2β_ (h)	V_d(area)_ (L/kg)	Cl (mL/kg·min)	AUC (ng·h/mL)	F (%)
**NDON**	6	28.76 ^a^	4.85	2.50	6.06	1.68 ^a^	12.54^a^	343.04 ^a^	98.62 ^a^
**SDON**	6	25.72 ^a^	2.76	1.02	5.63	1.98 ^a^	14.81^a^	280.51 ^a^	89.26 ^a^
**MK37**	4	8.16 ^b^	3.38	1.92	3.00	0.56 ^b^	7.52^b^	38.43 ^b^	9.46 ^b^
**MM37**	4	6.32 ^b^	3.40	1.63	4.43	0.54 ^b^	5.50^b^	35.98 ^b^	9.28 ^b^
**MK79**	5	4.47 ^b^	3.03	1.06	6.43	0.69 ^b^	4.90^b^	15.08 ^b^	4.40 ^b^
**MM79**	5	4.19 ^b^	2.26	0.65	6.21	0.93 ^b^	6.68^b^	22.34 ^b^	6.15 ^b^
*p* value		<0.0001	0.373	0.400	0.043	<0.0001	<0.0001	<0.0001	<0.0001

Note: Letters a and b indicated significance *p* < 0.05.

## 3. Discussion

### 3.1. Effects of Intravenous Application of DON and DONS (DON_iv_, DONS_iv_)

The administration of pure DON at the indicated dose of 50 µg/kg BW induced sickness and emesis within minutes and was characterized by individual variation which agrees with earlier findings of Goyarts and Dänicke [10]. In that study, the initial concentration *A* + *B* of DON at *t* = 0 ranged between 46.2–139.9 ng/mL plasma and was consequently lower than the corresponding levels of 120.7–708.5 ng/mL as determined in the present experiment. However, the high concentration of animal IV2 could be the result of the lower goodness-of-fit. The total plasma clearance of both studies was comparable (3.61 *vs.* 3.81 mL/kg·min). Some differences in kinetic profiles as observed in both experiments might be based, among other things, on the different genetic backgrounds of the pigs. The apparent volume of distribution of 0.61 L/kg after DON_iv_ was in accordance with the volume of total body water reported by Greenblatt and Shader [11]. So it could be assumed that DON distributed among the complete body water and so very high plasma concentrations were observed after injection. In comparison, kinetics after oral administration differed obviously and could be explained by the absorption rate and time after oral ingestion as well as the hepatic “first pass” effect [10]. That meant DON-contaminated feed reached the stomach and intestinum, DON was resorbed in the hepatic portal system and firstly entered into the liver. Here, the liver metabolized DON into DON metabolites to a certain extent before it reached the rest of the circulatory system. On this way, the bioavailability after oral administration could be reduced in comparison to intravenous injection.

With regard to IV application of DONS, pigs did not show any clinical effects. In plasma, all DONS and additional DON could be detected. The DON plasma level after DONS_iv_ reached a maximum (*A* + *B*) of 17.33 ng/mL (Table 4). As discussed by Schwartz *et al.* [9], DONS 3 is unstable and degraded mainly to DON, followed by DONS 1 and 2 at pH values > 5, as well as at higher temperature (37 °C) for 24 h or upon storage for a few days at 22 °C. Therefore, DON detection could be explained by DONS 3 conversion at the prevailing physiological conditions. 

In interpreting the results it needs further to be taken into account that the preparation of the injection solution with physiological saline (0.9% NaCl solution) from the standard solution of the DONS mixture (stabilized with 1% formic acid) was associated with distinct changes in the pattern of particular DONS, probably attributable to pH-changes resulting in a marked decrease of the DONS 3 proportion. Due to the pH value of 5.17 in NaCl solution, as well as the overnight storage at 4 °C, changes in DONS pattern before application were induced. The corresponding pH values in the standard and injection solution amounted to 1.56 and 2.66, respectively. The determination of concentrations in bolus for IV application resulted in a marked reduction of DONS 3, a substantial increasing of DONS 1, and the formation of DON (Figure 2, inserted table). Due to known structural instability of DONS 3 [9], it could be concluded that DON, and also DONS 1, was mainly formed from DONS 3 in bolus solution. DONS 1 and 2 were more stable than DONS 3 and so they could be detected in plasma of pigs. The concentration of DON after DONS_iv_ was much too low in that it would cause adverse effects in pigs.

When the DONS bolus was applied to pigs, the acidic pH value of 2.66 could be buffered by different buffer systems to keep a consistent pH level of 7.41 (average) in blood [12]. DONS 3 is not stable under physiological pH range between 7.35 and 7.45. From this point of view, the DON occurrence, as well as DONS ratio in plasma, could be substantiated (Figure 2). The dependence of the DONS stability on the pH value was already mentioned by Young [13] and Beyer *et al.* [14]. However, at that time, only two compounds were described, specified as 8-DONS and 10-DONS. They differ in their structural characteristics but have the same molecular mass. One substance, 10-DONS was predominantly formed due to reaction of DON and sodium-metabisulfite (SBS). A recent study by Schwartz *et al.* [9] showed that 10-DONS was one compound of DONS 3 which occurs as the keto form, but there is also a hemiketal form whereby both compounds exist in equilibrium. Additionally, two further DONS compounds (DONS 1 and 2) were detected. The stability of these recently discovered substances differs in dependence on pH value as a key factor but also depends on other factors such as temperature and storage time.

### 3.2. Oral Administration of DON-Containing Diet, either Untreated (NDON) or Dry Treated (SDON) with Sodium Sulfite

The oral administration of a DON bolus of 75 µg DON/kg BW corresponded to a dietary DON concentration of 6 mg DON/kg (NDON). DON was detected in plasma 15 or 30 min after giving the bolus. Therefore, a rapid absorption in the upper digestive tract might be deduced. Dänicke *et al.* [15] suggested that almost 90% of orally-administered DON was absorbed in the stomach and the first third of the small intestine. The total DON bioavailability of 98.6% ± 23.6% after a single dose was slightly higher than 91.5% ± 27.4% reported by Goyarts and Dänicke [10] which might be caused by the DON-containing matrix. In our present study, maize served as contaminated feedstuff instead of wheat. In various studies, the effect of DON source, including both different DON-contaminated feedstuffs and pure added DON, played a role in interpreting the observed effects. Friend *et al.* [16] demonstrated the difference between DON-contaminated wheat and maize in pig nutrition, whereby adverse effects on pigs after feeding the DON-containing maize diet were much more pronounced than after wheat feeding. This aspect might explain the higher bioavailability as well as the higher DON concentrations in plasma after oral consumption of DON-contaminated maize compared to DON in wheat. This is evidenced by maximum concentration c_max_ of 28.76 ng/mL compared to 15.2 ng/mL [10]. The time t_max_ reaching the maximum concentration differed markedly between 1.65 h and 4.85 h in the present study but it was reducible to animal O-5 because the kinetic was different to other pigs of this group.

The intention for *in vivo* detoxification is to initiate an immediate reaction to degrade DON in the limited period of time when contaminated feed is passing as ingesta through the digestive tract together with a detoxifying agent [15]. However, the promising approach to supplement dry Na_2_SO_3_ to the negative control diet immediately before feeding did not achieve DON reduction in blood as deduced from results of a previous *in vitro* study [8] demonstrating that the addition of Na_2_SO_3_, water,, and propionic acid to DON-contaminated maize initiated an immediate reaction in which DON was reduced and DONS were formed in the so-treated maize. The initial concentration of DON c_0_ in blood pointed out that only a marginal reduction from 48 ng/mL to 40 ng/mL took place and also the maximum concentration in plasma (c_max_) of 25.72 ng/mL confirmed this statement. Although approximately 89% of the applied DON dose was recovered in the form of DON, we wanted to exclude the possibility that DONS was formed by the dry treatment, but not absorbed. Therefore, we examined the principal absorbability of DONS by adding a DONS mixture to an uncontaminated (and untreated) diet. Similarly to intravenous DONS bolus, the DONS/distilled water mix was examined for pH which amounted to 2.81 so that no changes in DONS pattern should be expectable. However, the measurements of pure DONS_po_ solution resulted in changes of proportions of DONS 1, 2, and 3 and the formation of DON (Figure 4). Only DON and, rarely, traces of DONS 1 and 2 (<LOQ) could be detected in plasma. This result might be an indication of the poor absorption properties of DONS. Together with the ineffective DON reduction in plasma, an *in vivo* detoxification by adding dry Na_2_SO_3_ to a contaminated diet seems to be inefficient.

### 3.3. Effects of Wet Preservation Method with Sodium Sulfite

When feeding the wet preserved and with sodium sulfite-treated DON-contaminated maize, the stability of the formed DONS could be proven by corresponding low DON plasma concentrations. Although no DON was detected in treated maize samples, minor plasma DON levels were detected showing kinetics similar to the NDON variant but at a much lower level. Different DONS formed during preservation in feed could not be detected in plasma with the exception of a few pigs (MK_79_/MM_79_) where DONS 2 was found at low concentrations (Figure 5). Compared to study by Dänicke *et al.* [7], SBS treated triticale fed to piglets resulted in a verifiable DON reduction as well as detectable content of DON sulfonate in plasma (15.5 ng/mL). However, only one DONS compound was known at that time. In contrast to our study, it had to be taken into account that piglets were fed over a period of 28 days while a single dose was administered in our study. As mentioned above, Schwartz *et al.* [9] described important influence factors on DONS formation and stability. During the period of preservation, the DONS pattern changed extremely. It started with a high DONS 3, increasing DONS 2 and less DONS 1 concentration after a few minutes, changing towards low DONS 3, high DONS 2 and slight rising DONS 1 concentration after 79 days of storage [8]. This meant that a stable relation between compounds was reached at that time. When mixing the preserved maize to basal diet with an addition of water, the matrix effects and reaction conditions could change so that also further shifting in ratio might be possible. But especially after feed intake, the stability of DONS is dependent on pH value in digestive tract and therefore DONS 3 is supposed not to survive the neutral and slightly alkaline pH range in the small intestine as well as in blood [9]. From the detected DON levels in plasma, it was assumed that DON was predominantly formed from DONS 3. In this case, the release rate of DON from DONS is defined as bioavailability. The comparison of DON availability after negative control diet (6 mg DON/kg) with DON occurrence after feeding preserved diets clearly demonstrated our aim to reduce the DON bioavailability by the use of the wet preservation method with Na_2_SO_3_.

Furthermore, the fact that we could not, or only rarely, detect the different DONS compounds after oral ingestion of demonstrable existing DONS in feed could suggest a minor absorption from digestive tract into blood. An indication for this assumption was the evidence of DONS 2 traces (c_DONS2_ = 1.1 ng/mL DON equivalents) in plasma of pigs fed with MK_79_/MM_79_. These two variants had high DONS 2 and low DONS 3 concentration at the end of preservation duration [8] which represented the more stable relation. The lower DON concentration of 4.33 ng/mL compared to variants MK_37_/MM_37_ with 7.24 ng/mL substantiated the advantageous effect of a longer preservation period. After 37 days, relatively more DONS 3 to DONS 2 occurred.

## 4. Experimental Section

### 4.1. Experimental Design and Treatments

The experiment was designed as a kinetic study. The treatments included the administration of a single bolus, either po with feed serving as vehicle, or IV with standards dissolved in physiological saline (DON, DONS). Due to the instability of DONS 2 and 3 upon evaporation to dryness, the standards of the three DONS were dissolved in water acidified with 2% formic acid. The po-treatments included untreated DON-contaminated maize meal (NDON); DON-contaminated maize meal to which 5 g Na_2_SO_3_ was added dry (SDON); uncontaminated maize meal to which a mixture of DONS was added (DONS_po_); DON-contaminated maize meal (MM); and kernels (MK) wet preserved with Na_2_SO_3_ for 37 (MM_37_, MK_37_) and 79 (MM_79_, MK_79_) days. The intention of these po treatments was to compare various toxicokinetic variables after administration of boli which corresponded to comparable diet concentrations of either DON (NDON, SDON) or DONS (DONS_po_, MM_37_, MK_37_, MM_79_, MK_79_) (see Table 8). Differences in blood profiles of DON, DONS 1, DONS 2, and DONS 3, and in related toxicokinetics, including bioavailability, would therefore enable conclusions to be drawn on the stability of DONS *in vivo*. In order to allow estimation of bioavailability it was necessary to obtain the plasma kinetics after IV administration of DON and DONS (DON_iv_, DONS_iv_, Table 7).

A total of 16 castrated male pigs, crossbred German Landrace × Pietrain, were used for these kinetic experiments. After an adaption phase to the balance cages, eight animals underwent surgery per trial. Animals were equipped with two permanent intravenous catheters in the external *vena jugularis* for serial blood sampling [10]. After surgery the animals were allowed to recover for one day. On test day, zero blood samples had to be collected from pigs before exposure. Zero blood samples served as reference values to which the data after oral or intravenous bolus related. Serial blood samples (10 mL) were drawn prior to feeding and after 15, 30, 60 and 90 min and then after two, three, four, six, eight, 10, 12, and 24 h for pigs receiving compounds by oral administration. For pigs dosed intravenously, shorter time intervals were chosen at the beginning: five, 10, 15, 20, 30, 45 min and then following the same intervals as for oral exposure. For a clear classification in result Table 1, Table 2, Table 3, Table 4, Table 5 and Table 6, we differentiated between IV- (intravenous bolus) and O- (oral bolus) animals. Due to repeated use of individuals it was numbered continuously. 

**Table 8:** List of investigated treatments.

Treatment	Route of Administration	Description	Bolus	Na_2_SO_3_ Addition	Mean Dose (µg/kg BW)	Mean Diet Concentration (µg/kg or µg/kg DON Equivalents)	Abbreviation	*n*
1	po	DON from untreated contaminated maize (negative DON control)	DON	-	70	6000	**NDON**	6
2	po	DON from untreated contaminated maize supplemented with dry Na_2_SO_3_	DON	5 g/kg	70	6000	**SDON**	6
3	po	DONS from treated contaminated maize kernels after 37 days of preservation	DONS	10 g/kg	DONS 1 = 3 DONS 2 = 67 DONS 3 = 55	DONS 1 = 184 DONS 2 = 3551 DONS 3 = 2935	**MK_37_**	4
4	po	DONS from treated contaminated maize meal after 37 days of preservation	DONS	10 g/kg	DONS 1 = 4 DONS 2 = 94 DONS 3 = 21	DONS 1 = 227 DONS 2 = 3845 DONS 3 = 2748	**MM_37_**	4
5	po	DONS from treated contaminated maize kernels after 79 days of preservation	DONS	10 g/kg	DONS 1 = 4 DONS 2 = 60 DONS 3 = 43	DONS 1 = 253 DONS 2 = 5884 DONS 3 = 1293	**MK_79_**	5
6	po	DONS from treated contaminated maize meal after 79 days of preservation	DONS	10 g/kg	DONS 1 = 5 DONS 2 = 91 DONS 3 = 15	DONS 1 = 184 DONS 2 = 6078 DONS 3 = 977	**MM_79_**	5
7	po	pure DONS from standard solution	DONS	-	100	3000	**DONS_po_**	4
8	IV	pure DON from standard	DON	-	50	-	**DON_iv_**	5
9	IV	pure DONS from standard solution	DONS	-	50	-	**DONS_iv_**	6

Blood samples were centrifuged to separate the plasma and kept frozen at −20 °C until analysis. Sodium heparin (2 mL/750 mL normal saline) was used to keep the catheters functioning. Treatments and experiments were approved according to § 8 of the Animal Welfare Act of 18 May 2006 and carried out following the guidelines of the Lower Saxony State Office for Consumer Protection and Food Safety, Germany.

### 4.2. Practical Procedures

Two experimental diets were produced in the feed mill of the experimental station of the Friedrich-Loeffler-Institute in Braunschweig, Germany (Table 9). The diets were formulated to meet or exceed all nutritional requirements for fattening pigs as recommended by the German Society of Nutrition Physiology (GfE, 2006) [17]. During the whole experimental period, a restricted feed regime was implemented. The daily feed amount of 1.0 kg in Trial 1 and 1.4 kg in Trial 2 was given in two equal portions at 7:00 am and 1:00 pm. The difference in feed amount was explained by various body weights of animals in the first trial. For evaluation of the results we wanted to achieve a complete feed consumption and so we defined the amount of diet. In the second trial there was no problem. Animals were fed with the control diet, apart from the morning feeding on test days, whereby animals received their oral bolus. DON-contaminated maize used in the study had previously been cultivated on the experimental field of the Institute of Animal Nutrition of the Friedrich-Loeffler Institute in Braunschweig, Germany, and had been inoculated with spores of *Fusarium graminearum*. The initial concentration of DON in maize amounted to 45.2 mg/kg. Due to the defined DON concentration of 6.0 mg/kg for the negative control group (NDON), the proportion of contaminated maize bolus was 13.3% in diet. Hence, the DON-contaminated diet comprised 6.0 ± 0.7 mg DON/kg diet, whereas the control diet contained 0.21 ± 0.02 mg DON/kg.

**Table 9:** Composition of the experimental diets (g/kg, based on a dry matter content of 88%).

Ingredients	Experimental Diet	Control Diet
Barley	389	350
Wheat	310	279
Maize	0	100
Soybean meal	244.4	220
Soybean oil	16.7	15
Premix *	33.3	30
Lysine-HCl	4.4	4
DL-Methionine	1.1	1
L-Threonine	1.1	1
Analysed composition		
Crude protein	198.41	189.19
Deoxynivalenol (mg/kg)	6.0 ± 0.7	0.2 ± 0.02

* Provided per kg of diet: Ca 6.1 g; P 1.5 g; Na 1.4 g; Mg 0.3 g; Fe 100 mg; Cu. 25 mg; Mn 50 mg; Zn 100 mg; I 1.3 mg; Se 0.4 mg; Co 0.5 mg; vitamin A 10,000 IU; vitamin D3 1000 IU; vitamin E 30 mg; vitamin B1 18.8 µg; vitamin K3 1.3 mg; nicotinic acid 12.5 mg; pantothenic acid 8.4 mg; choline chloride 125 mg.

Two different injection solutions were prepared for IV administration. Standard DON (Sigma D-0156, Deisenhofen, Germany), present in solid form, was dissolved in physiological saline to prepare a 250 µg/mL stock solution. After weighing, an individual amount was drawn up into syringes with a dose of 50 µg/kg BW. The concentrations of DONS 1, 2, and 3 in DONS standard solution were 3240, 1720, and 7300 µg/mL, respectively. The production of DON sulfonates standard mixture was performed and described by Schwartz *et al.* [9]. Thereafter, the standard was mixed with physiological saline to prepare a 250 µg/mL DONS stock solution. The further handling was similar to DON.

For administration of DONS_po_ (100 µg/kg BW), the animals were weighed and the corresponding amount of DONS standard solution was mixed with 10 mL distilled water and added to diet immediately before feed consumption.

Due to known dependency of DONS stability on pH value, we compared the pH of standard and bolus solutions. The determination was carried out with a pH meter (WTW GmbH, Weilheim, Germany). Both an addition of physiological saline and distilled water caused changes in pH value, and so shifts in DONS ratio took place.

### 4.3. Analyses

Determination of DON in contaminated and control maize was carried out by high performance liquid chromatography (HPLC) with diode array detection (DAD) after clean-up with immune-affinity columns (IAC, VICAM, MA, USA) according to a modified VDLUFA method of Valenta *et al.* [18]. The detection limit of DON was 0.03 mg/kg.

Samples of the experimental diets were analyzed for DON and DONS by ultra high performance liquid chromatography (RP-UHPLC) coupled with tandem mass spectrometry (UHPLC-MS/MS) according to Schwartz-Zimmermann *et al.* [19]. The quantification limits (LOQ) of DONS 1, 2, and 3, as well as DON were 0.2, 0.4, 0.4, and 0.5 mg/kg, respectively. The corresponding recoveries were approximately 103%, 94%, 108%, and 108%, respectively.

For quantitative determination of DONS 1, 2, and 3 in plasma, an HPLC-MS/MS analysis was conducted on an Agilent 1200 (Agilent Technologies, Böblingen, Germany) series HPLC system coupled to a 4000 QTrap mass spectrometer (AB Sciex, Foster City, CA, USA). Compounds were separated in gradient elution on a Phenomenex Kinetex 5u C-18 column (150 × 2.1 mm, 5 µm, 100 A) at 30 °C using water and methanol, both containing 0.1% formic acid (*v/v*), as mobile phases A and B. The flow rate was 300 µL/min, the injection volume 5 µL and the total run time was 17 min. The retention times of DONS 1, 2, and 3 were 2.31, 4.71, and 5.88 min, respectively. Tandem mass spectrometric detection was carried out in negative ion mode after electrospray ionization at 600 °C (ion spray voltage: −4500 V). The transitions used are given in Table 10. The method was validated in-house for all analytes based on the guidelines of the FDA and European community. Calibration graphs (0.3–480 ng/mL) were prepared and good linearity was achieved (*r* ≥ 0.99). The recoveries of DONS 1, 2, and 3 averaged 116% ± 2%, 78% ± 3%, and 70% ± 3%. The limits of detection in plasma amounted to 0.48, 0.34, and 1.21 ng/mL and the corresponding limits of quantification were 1.59, 1.14, and 3.99 ng/mL, respectively.

Analysis of DON concentration in plasma was performed based on a method according to Brezina *et al.* [20]. The LOD amounted to 0.22 ng/mL and the LOQ was 0.72 ng/mL.

**Table 10:** Transitions of DONS 1, 2, and 3 used for mass spectrometric detection.

Analyte	Q1	Q3	Time [msec]	DP [V]	CE [V]	CXP [V]	Retention time [min]	LOD [ng/mL]	LOQ [ng/mL]
DONS 1	377.1	79.9	150	−120	−82	−3	2.31	0.48	1.59
DONS 2	377.1	81.0	150	−70	−52	−3	4.71	0.34	1.14
DONS 3	377.1	79.9	150	−55	−96	−1	5.88	1.21	3.99

DP = declustering potential; CE = collision energy; CXP = collision cell exit; LOD = limit of detection; LOQ = limit of quantification.

### 4.4. Toxicokinetic and Statistical Analysis

Plasma concentration data after oral administration variants were fitted to the Bateman function [21] (Equation (1)):

(1) $$c_{t}=\frac{{Dk}_{a}}{V\left(k_{a}-k_{el}\right)}\cdot \left(e^{-k_{el}\cdot t}-e^{k_{a}\cdot t}\right)$$

where *c_t_* is the DON concentration at time *t*. *D* is the systemic dose, *i.e.*, the oral dose multiplied with the bioavailability F, of DON/DONS (µg/kg body weight), *V* the apparent volume of distribution (L/kg), *k*_a_ the absorption constant, *k*_el_ the elimination constant, respectively, and *t* the time after the bolus (h). After extrapolation of the elimination curve to *t* = 0 the DON concentration $c_{0}=\frac{D}{V}$ was determined as a hypothetical IV dose that would occur after a complete absorption of the oral dose (Garrett, 1994) [22]. The maximal plasma concentration (*C*_max_) and the times at peak concentrations (*t*_max_) were estimated numerically based on the individually fitted curves.

A two-compartment model according to Prelusky *et al.* [23] was used after intravenous application of DON and DONS:

(2) $$c_{t}=A\cdot e^{-\alpha \cdot t}+B\cdot e^{-\beta \cdot t}$$

where *c_t_* is the DON/DONS concentration at time t. *A* + *B* is the initial concentration at *t* = 0, *i.e.*, C_0_, α, and β are the rate constant of distribution or elimination phase, respectively, and t the time (h). The non-linear curve fitting module of the Statistica for the Windows^TM^ operating system (StatSoft Inc., Tulsa, OK, USA) was used to fit the data to Equations (1) and (2).

Further kinetic parameters were derived from the fitted functions as also described by Prelusky *et al.* [23]. The half-life was calculated as $t_{1/2\alpha }=\frac{ln\;\left(2\right)}{k_{a}\;or\;\alpha }$ for absorption or distribution phase and $t_{1/2\beta }=\frac{ln\left(2\right)}{k_{el}\;or\;\beta }$ for the elimination phase, respectively. The total area under the plasma concentration-time curves (AUCs) for DON/DONS was determined by using the trapezoidal rule method over the interval from zero to 24 h after the bolus. The apparent volume of distribution by area (*V*_d(area)_) following intravenous application was calculated by $V_{d\left(area\right)}\;=\;\frac{D}{AUC}\cdot \frac{t_{1/2\beta }}{\ln\left(2\right)}$ as an indicator of V_d_ corresponding to the elimination phase and thus covering large parts of the kinetics. The plasma clearance (Cl) of DON was estimated by $Cl=\frac{ln\left(2\right)\cdot V_{d\left(area\right)}}{t_{1/2\beta }}$.

Systemic bioavailability (F) of orally administered DON/DONS was principally estimated based on the ratios of the corresponding AUCs and expressed in % of the corresponding AUC after IV administration. As just DON was detectable in systemic blood after administration of the oral DON or DONS bolus (either from standards or from contaminated/preserved maize) only the AUC_po_ corresponding solely to DON was used and divided by the AUC_iv_ corresponding to the IV DON-bolus. In addition, in order to evaluate the decomposition of the IV administered DONS mixture the DON-AUC was divided by the sum of individually estimated AUCs for DONS 1 and DONS 2 and declared as F. In each case, the AUCs were corrected for the administered dose. 

Important kinetic parameters estimated after oral administration were subjected to analysis of variance (ANOVA) with the treatment group as the fixed factor. In case of significant mean value differences particular significance was identified using post-hoc multiple LSD-test. The significance level was set at *p* ≤ 0.05.

All statistics were carried out using the Statistica for Windows^TM^ operating system (StatSoft Inc., Tulsa, OK, USA).

## 5. Conclusions

Generally, the wet preservation method with Na_2_SO_3_ for contaminated maize significantly reduced the bioavailability of DON in pigs. The reduction of DON in feed was confirmed by low DON plasma levels after oral intake suggesting stability of DONS formed during preservation. The three reaction products, DON sulfonate 1, 2, and 3, need to be considered individually because of their different stability characteristics. Due to instability of DONS 3 and re-conversion to DON under physiological conditions, a longer preservation time should be recommended because of the long-lasting process to form a stable relation between the DONS compounds. Thereafter, DONS 3 concentration should be as low as possible. All in all, the wet preservation with Na_2_SO_3_ is an efficient procedure for decontamination whereby adverse impacts on pigs could be avoided. Transfer to practice should be possible when necessary conditions for feed preservation would be observed. Furthermore, the use of preserved contaminated cereals could also prevent economic losses. The supplementation of (dry) Na_2_SO_3_ immediately before feeding had only an insufficient DON-reducing effect and is, therefore, unsuitable for *in vivo* detoxification. The IV application of DONS as mixture demonstrated the dynamics of different DONS in the circulation. After oral administration, the DONS formed could not, or only hardly, be detected in plasma. On this basis, further studies should examine higher initial DONS concentrations in feed, as well as a longer exposure time. Additionally, these tests are needed to investigate more closely the absorption, metabolism, and excretion of DONS, as well as the analysis of residues in other physiological specimens.
